# Editorial to introduce new section in journal “null results”

**DOI:** 10.1186/s12986-024-00796-x

**Published:** 2024-04-24

**Authors:** Barbora Piknova, Mario Siervo

**Affiliations:** 1 Molecular Medicine Branch, NIDDK, NIH, Bethesda, MD USA; 2 School of Life Sciences, The University of Nottingham Medical School, Queen’s Medical Centre, Nottingham, UK

Dear authors, editors, and readers!

Even if it already March, year 2024 is still young. With every year passing, our journal grows better and stronger. Let us share some statistics from the past year to support this claim– after all, we are all scientists, our thinking is based on facts. In 2023 *we* received the highest number of submissions, 485, which is an increase from 417 received in 2022. The authors receive the first editorial notification in average in about 14 days and the whole editorial process from submission to acceptance is about 150 days. Majority– 80% of the published information was shared on social media and downloaded 95,000-times. Our impact factor in 2023 was 4.5, which is testimony to the excellent quality of published manuscripts.

Not only we have grown considerably in the number and quality of received and published manuscripts, our editorial board has also considerably grown in number, experience and expertise in multiple disciplines. We express our deepest gratitude to their commitment and hard work assuring the highest quality of our publications.

For the new year of 2024 we aim to further enlarge scopes of our journal, with an inclusion of new permanent section of the journal where we would like to publish results from your “good-evidence-based hypothesis, but Nature works differently” studies. Let us explain what is in this rather cryptic title. Every single scientist among us did experience (or sooner-later will experience) the “scratching-head” moment when, after formulating perfectly sound hypothesis, securing necessary funding and permissions, performing sometimes rather long study, and collecting data, we came to the moment of truth that “nothing happened, there is no effect”. Depending on subject and the size of “no effect”, such realization may prevent the publication of such work. We believe that well-designed and executed “no effect” study is as important for the public as study showing an effect, since it shows that previous hypothesis did not weight in some important factors, and it may lead to a novel hypothesis that advance science as whole. In addition, it could also prevent the un-necessary waste of time, material and, in case of animal studies, reduce the number of killed animals. Unfortunately, we have to admit that journals are generally less likely to accept a “negative result” (as this case is sometimes referred to) study. In Nutrition & Metabolism we decided to offer a permanent platform to authors to share their “no effect” studies in fields within the established scope of journal. We hope that the new section of our journal (which could be think of as “good-evidence-based idea, but Nature works differently”) will serve as an important database of “null results” for all scientists in field of nutrition and metabolism helping them to design better experiments and advance objectivity of science. In addition to all traditional sections of manuscript (from abstract through conclusions) we would like to encourage authors to write their thoughts about why they were surprised with the results they got and what are implications and significance of these results for the field. We believe that understanding why the sound hypothesis did not work is sometimes more important for the advancement of the field than the simple positive result.

Once again, we would like to thank to all of you helping us to grow our Nutrition & Metabolism journal better and bigger. We would like to hear from you more in the future and we hope you will entrust us to handle your exciting new manuscripts.

Barbora Piknova, PhD and Mario Siervo, PhD.

Editors in Chief.

## Data Availability

No datasets were generated or analysed during the current study.

